# Novichoks: The Dangerous Fourth Generation of Chemical Weapons

**DOI:** 10.3390/ijms20051222

**Published:** 2019-03-11

**Authors:** Tanos C. C. Franca, Daniel A. S. Kitagawa, Samir F. de A. Cavalcante, Jorge A. V. da Silva, Eugenie Nepovimova, Kamil Kuca

**Affiliations:** 1 Laboratory of Molecular Modeling Applied to the Chemical and Biological Defense (LMCBD), Military Institute of Engineering, Rio de Janeiro 22290-270, RJ, Brazil; kitagawa.daniel@ime.eb.br; 2 Center for Basic and Applied Research, Faculty of Informatics and Management, University of Hradec Kralove, Rokitanskeho 62, 50003 Hradec Kralove, Czech Republic; 3 Brazilian Army CBRN Defense Institute (IDQBRN), Brazilian Army Technological Center (CTEx), Rio de Janeiro 23020-470, Brazil; samir.cavalcante@eb.mil.br (S.F.d.A.C.); capalberto05@yahoo.com.br (J.A.V.d.S.); 4 Department of Chemistry, Faculty of Science, University of Hradec Kralove, Rokitanskeho 62, 50003 Hradec Kralove, Czech Republic; Evzenie.N@seznam.cz; 5 Philosophical Faculty, University of Hradec Kralove, Rokitanskeho 62, 50003 Hradec Kralove, Czech Republic

**Keywords:** Novichoks, binary weapon, nerve agents, chemical warfare

## Abstract

“Novichoks” is the name given to the controversial chemical weapons supposedly developed in the former Soviet Union between the 1970s and the 1990s. Designed to be undetectable and untreatable, these chemicals became the most toxic of the nerve agents, being very attractive for both terrorist and chemical warfare purposes. However, very little information is available in the literature, and the Russian government did not acknowledge their development. The intent of this review is to provide the *IJMS* readers with a general overview on what is known about novichoks today. We briefly tell the story of the secret development of these agents, and discuss their synthesis, toxicity, physical-chemical properties, and possible ways of treatment and neutralization. In addition, we also wish to call the attention of the scientific community to the great risks still represented by nerve agents worldwide, and the need to keep constant investments in the development of antidotes and ways to protect against such deadly compounds.

## 1. Introduction

Despite of all the eradication efforts made by the Chemical Weapons Convention (CWC) (https://www.opcw.org/chemical-weapons-convention/), recent episodes involving nerve agents have proved that this kind of chemical weapon (CW) is still far from controlled. Some examples are the use of sarin several times in the Syrian civil war, and the assassination of Kim Jong-Nam (half-brother of the North Korea dictator Kim Jong-Un) with VX in February of 2017 (in the Kuala Lumpur airport) [1]. Further, a new threat has emerged in this scenario after the recent attempted assassination of the former Russian spy Sergei Skripal and his daughter, Yulia, on UK soil [1]. According to the Organization for Prevention of Chemical Weapons (OPCW) (https://www.opcw.org/), the chemical used in this event was the controversial nerve agent known as novichok, considered the fourth generation of the CWs [2]. This name was given to the weapons created with some compounds of the A-series nerve agents and, supposedly, secretly developed in the former Soviet Union between the 1970s and 1990s. Because they were not included in CWC’s list of banned chemicals, the agents of this series were kept unknown from most of the scientific community, and little was done in developing ways of preventing or treating against intoxication with them. Its recent use in the UK presents a great opportunity for warning the scientific community about the risks they represent and urging the need to develop new antidotes as well as ways of detection, protection, and neutralization.

## 2. Development of the A-Series Nerve Agents

**Figure 1:**
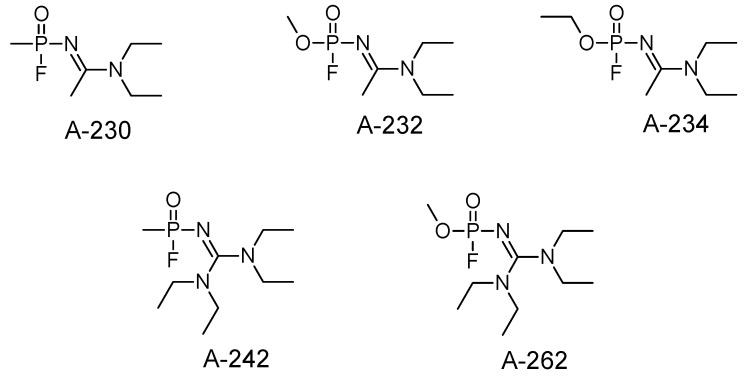
Structures reported by Mirzayanov for the A-series agents developed in the State Scientific Research Institute for Organic Chemistry and Technology (GOSNIIOKhT) by Petr Kirpichev [3].

In his book titled: “State Secrets: An inside chronicle of the Russian chemical weapons program” [3], the Russian dissident exiled in the United States, Vil S. Mirzayanov, denounces the former Soviet Union for having secretly developed novichok agents between the 1970s and 1990s. Mirzayanov is a former scientist who worked for 26 years at the State Scientific Research Institute for Organic Chemistry and Technology in Moscow, abbreviated in Russian language as GOSNIIOKhT (*Г*o*cH*ИИ*OXT* in Cyrillic). He claims to have witnessed, between 1971 and 1973, Petr Kirpichev and his assistants developing a new series of neurotoxic agents derived from the G-series and V-series agents, this was named as the A-series. According to his testimony, the first compound of this series synthesized and tested was *N*-2-diethylaminomethylacetoamidido-methylphosphonofluoridate (Figure 1). Essentially, it was a sarin derivative where the *O*-isopropyl group was replaced by the acetoamydin radical. This compound received the codename A-230 and was referred to as substance 84 in the internal reports of the GOSNIIOKhT. Agent A-230 presented a toxicity 5–8 times higher than the Russian VX (RVX), referred to as substance 33. However, it was found that it crystallized when stored at –10 °C [3]. This problem could be solved by adding *N*,*N*-dimethylformamide to the pure agent. After agent A-230, Petr Kirpichev and his group synthesized and tested the derivatives A-232, A-234, A-242, and A-262 (Figure 1). A-232 and A-234 (respectively the methoxy and ethoxy analogues of A-230) presented toxicity similar to RVX but were much more volatile and less stable in hydrolysis. A-242 and A-262 (the guanidine analogues of A-230 and A-232, respectively), were probably the first solid neurotoxic agents synthesized [3].

During the 1980s, A-230 passed all field tests, and in 1990 it was approved by the Soviet Army as a new chemical agent that could be used in all types of ammunition. Plans for mass production of this compound were then started, and a new CW production factory was designed in Pavlodar, Kazakhstan [3]. Funding for this project, however, disappeared with the collapse of the Soviet Union, and A-230 was never mass produced despite the experimental quantities (a few tons) were synthesized in the GOSNIIOKhT branches of Volsk and Volgograd [3]. Soon after A-230, A-232 also passed field tests and was incorporated to the Soviet Army’s arsenal. This agent had the advantages of being resistant to cold temperatures, and it circumvented the list of chemical agents controlled by the CWC because it belonged to the series of phosphoramidofluoridates. It was similar to the majority of the organophosphates (OPs) used in agriculture (pesticides and herbicides). OPs are not included in the schedules of CWC because, so far, all known OPs used as chemical weapons are phosphonates. As A-232 and A-234 are not phosphonates, they were ideal to cheat the CWC inspectors [3].

## 3. Binary Weapons

**Scheme 1:**
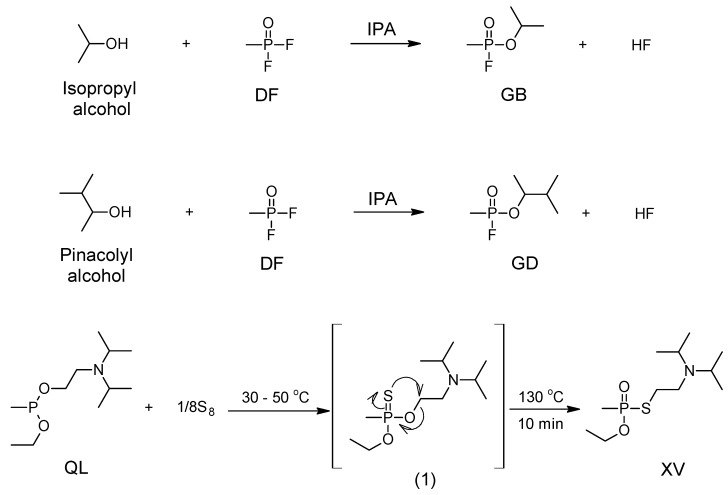
Production reactions of sarin (GB), soman (GD), and XV in binary weapons [5,6,7].

In chemical warfare, a binary weapon is defined as a CW where the toxic agent is produced during the flying time of the ammunition (rocket, missile, or grenade) towards the target [4]. The purpose of this kind of CW is to reduce the risks in the production, storage, transport, and even destruction of the toxic agent. This is possible because the starting materials are non-toxic (or less toxic) precursors, which are mixed to produce the chemical agent only after the ammunition is launched. For this task to be feasible the reaction has to be controlled to avoid overheating or explosion during the flight. Also, it should preferably run without solvent and has to be completed, with a high yield, in a matter of seconds. Usually the body of a binary projectile contains two separate canisters, one behind the other, each containing one starting material. The force of launching causes the breaking of the canisters, which mixes the starting materials and triggers the reaction to produce the chemical agent. The idea of designing this kind of ammunition originates from World War II [4], when military chemists developed, but never used, a binary bomb to deliver the blood agent arsine (AsH_3_). The blister agent *N*-(2-chloroethyl)-*N*-nitrosocarbamate (code named KB-16) was also considered as a potential binary weapon in the 1940s, but never fully developed. The first nerve agents developed as binary weapons were GB-2, GD-2, and VX-2, the binary versions of sarin, soman, and VX, respectively. They were all developed by the USA during the Cold War [5,6,7] and were considered the third generation of CWs [2]. GB-2 and GD-2 can be produced from the reaction between methylphosphonic difluoride (DF) and isopropyl or pinacolyl alcohol in an isopropylamine (IPA) solution (Scheme 1). VX-2 is produced from the reaction of *O*-Ethyl *O*-2-diisopropylaminoethyl methylphosphonite (QL) with elemental sulfur to produce the intermediate (1) that quickly rearranges to VX (Scheme 1).

**Scheme 2:**
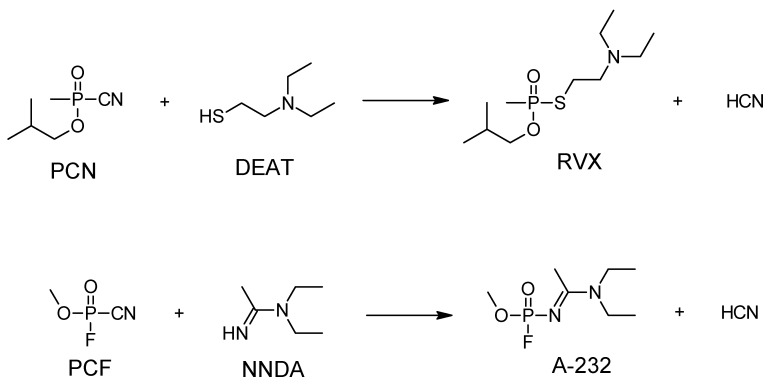
Production reactions of A-232 and substance 33 in novichoks # and 5 [3].

In response to the USA binary program, the Soviet Union started the FOLIANT program in the 1980s [8,9], with the goal of also developing this kind of CW. According to Mirzayanov [3], under this program, researchers from the GOSNIIOKhT only successfully weaponized A-232 and substance 33 as binary weapons, both in 1990. However, other sources in the literature mention that A-234 was also weaponized, as novichok 7 [2,10,11]. Binary A-232, weaponized as novichok 5, is reported as the product of the reaction between the methyl phosphorocyanidofluoridate (PCF) and *N*,*N*-diethyl-2-iminopropan-1-amine (NNDA) (Scheme 2). Binary substance 33, weaponized as novichok # [10], is produced from the reaction between 2-methylpropyl methylphosphonocyanidate (PCN) and 2-(diethylamino)ethanethiol (DEAT) (Scheme 2). Despite reporting the precursors, neither Mirzayanov nor other literature sources give any reactional details of these binary weapons. Mirzayanov also reported that, ironically, the former President of the USSR, Mikhail Gorbachev, who won the Nobel Peace Prize in 1990, signed the secret documents on 23 September 1989 that authorized the implementation of the project to transform A-232 into a binary weapon. Almost immediately after, the USA and USSR governments signed the Wyoming Memorandum of Understanding regarding CW disarmament [3].

## 4. Other Possible Chemicals in the A-Series

**Figure 2:**
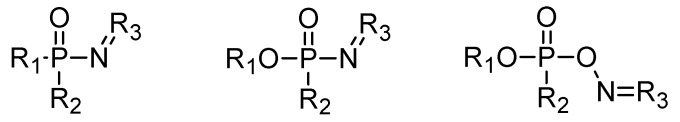
Possible scaffolds for the A-series nerve agents already reported in the literature [11].

**Figure 3:**
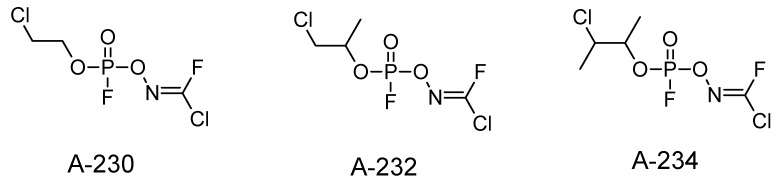
Possible A-series nerve agents, as reported by Hoenig [12].

**Figure 4:**
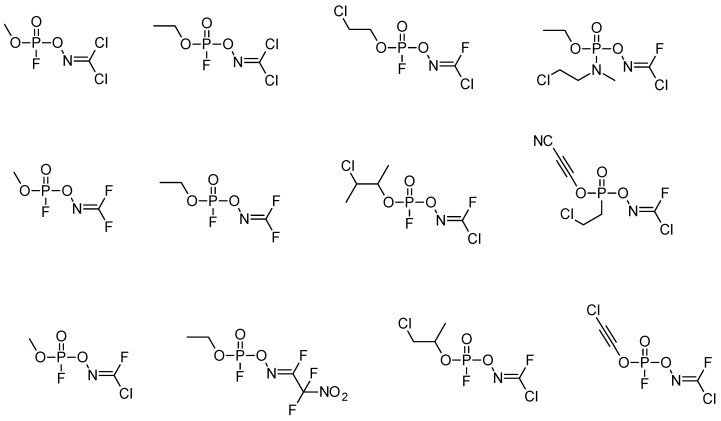
Possible structures of A-series nerve agents, as reported by Ellison [14] and Patocka [13].

Due to the high level of secrecy surrounding the FOLIANT program, very little is known about the real structures of the A-series nerve agents, and many speculative structures have been reported in the literature [3,11,12,13]. There is some consensus that all compounds of the A-series should be derivatives of one of the three scaffolds reported by Chai et al. [11], as shown in Figure 2. However, there is much speculation about the possibilities for the groups R_1_, R_2_, and R_3_. Different from Mirzayanov [3], Hoenig [12] speculates that compounds A-230, A-232, and A-234 are fluorophosphonates, as shown in Figure 3, where the N atom is not directly bound to the P atom, and they contain several halogens in the structure. Similar versions of these compounds were also reported by Ellison [14] and Patocka [13] (Figure 4).

It is very difficult to confirm if the structures reported in Figure 2 are the authentic scaffolds of the A-series nerve agents. This is due not only to the high level of secrecy surrounding these compounds, but also to the possibility of many of these compounds being fake structures intentionally leaked by counter intelligence services. Currently, the CWC recognizes the *O*-alkyl alkylphosphonamidates (Figure 2) as possible A-series derivatives, and classifies them as schedule 2.B.04. However, there is no detailed information about this series of OP compounds. Comparing to existing Schedule I nerve agents listed in the CWC, these compounds can be regarded as cholinesterase inhibitors. Therefore, exposure to them may lead to the SLUDGEM (Salivation, Lachrymation, Urination, Defecation, Gastrointestinal, Emesis, and Miosis) Syndrome [15].

## 5. Synthesis of A-series Nerve Agents

Discussion about the A-series nerve agents must be done carefully. Those compounds have not been confirmed yet, and most of the information available today is speculative or comes from questionable sources. One relevant aspect of the proposed synthetic route is related to the idea behind the development of these agents. As they have been planned to be prepared from chemicals not prohibited by the CWC, precursors should not be under the scrutiny of the OPCW at that time. Therefore, they could be disguised as other compounds, with lower toxicity and with fair use as pesticides. The other issue is that they may be weaponized as binary agents, being delivered on-site, and handled with safety.

**Scheme 3:**
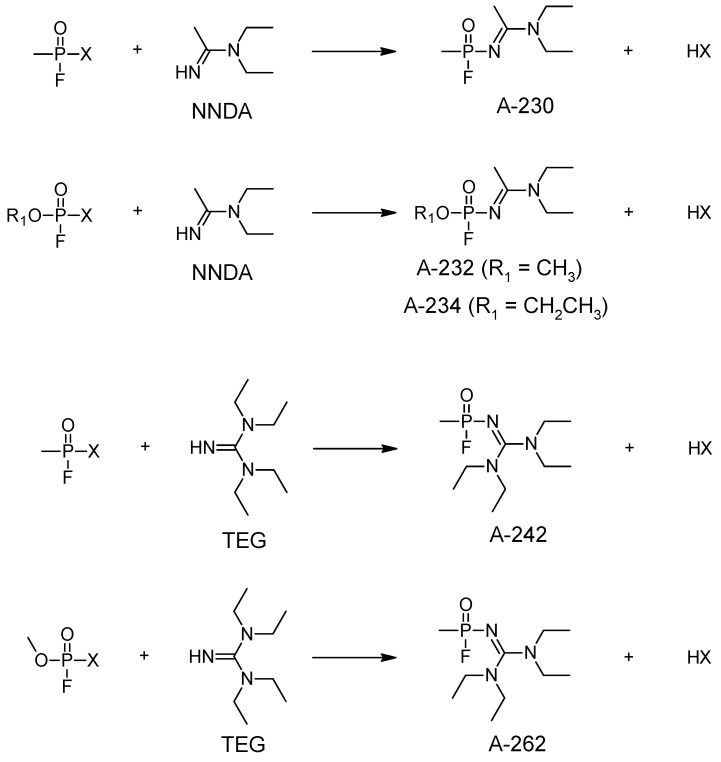
Possible synthesis routes for the A-series based on Mirzayanov’s report [2]. The leaving group X is either fluorine (F) or cyanide (CN).

According to Mirzayanov [3], A-230 can be synthesized following a synthetic route similar to the one used to obtain sarin and soman, where an amydine (instead of an alcohol) is condensed with DF (see Scheme 3). If *O*-methyl phosphonyldifluoride or *O*-methyl phosphonylfluoro cyanide is used instead of DF, A-232 can be obtained, while A-234 comes from the reaction with *O*-ethyl phosphonyldifluoride or *O*-ethyl phosphonylfluoro cyanide. A-242 and A-262 on this turn, can be obtained if the amydine is replaced by a 1,1,3,3-tetraethylguanidine (TEG) in the reactions to produce A-230 and A-232, respectively (see Scheme 3). As they are fluorophosphonates, A-232, A-234, and A-262 can be easily synthesized from the non-scheduled compounds *O*-ethyl phosphonyldifluoride and *O*-methyl phosphonyldifluoride and, therefore, circumvent the CWC.

**Scheme 4:**
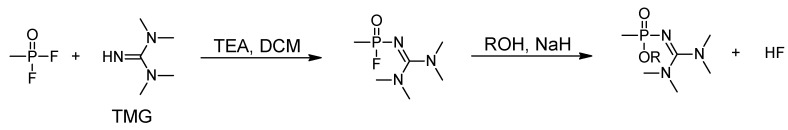
Microsynthesis of *O*-Alkyl *N*-[bis(dimethylamino)methylidene]-*P*-methylphosphonamidates, as reported by Hosseini et al. [16].

For analytical purposes, and to contribute to the OPCW Central Analytical Database (OCAD), in the development of ways of detection for the A series, Hosseini et al. [16] have reported a microsynthesis of a series of *O*-alkyl *N*-[bis(dimethylamino)methylidene]-*P*-methylphosphonamidates. They replaced TEG by 1,1,3,3-tetramethylguanidine (TMG), and extended the synthetic route of A-242 through an additional reaction of the final product with the appropriate alcohol in the presence of NaH (see Scheme 4).

**Scheme 5:**
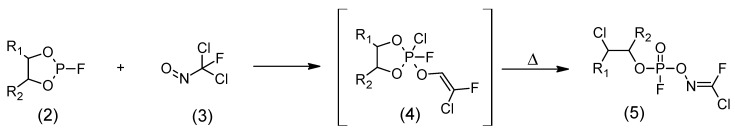
Synthetic route proposed by Hoenig [12] for the synthesis of the A-series nerve agents.

Different from Mirzayanov [3], Hoenig [12] hypothesized that their alleged A-series compounds can be synthesized following the two-step route shown in Scheme 5. According to him, starting from a di-substituted fluorophosphite (2), the nitroso form of *O*-chlorodihaloaldoxime (3) (which is structurally related to phosgene) acts as a nucleophile, affording a dihalophosphite intermediate (4). Heating leads to a rearrangement to the fluorophosphate derivatives (5), which he claims to be the scaffold of the A-series. Some authors speculate that the novichoks are in fact the intermediates (4) or precursors of derivatives (5) [12,17].

## 6. Physico-Chemical Properties of A-series Nerve Agents

Mirzayanov [3] reported that, like the other nerve agents, A-230, A-232, and A-234 are liquids, while A-242 and A-262 are the first nerve agents known to be in the solid state at room temperature. However, as there is virtually no information in the literature about this series of warfare agents, their physico-chemical properties are mostly estimated [2,3,7]. Table 1 reviews some properties collected from the literature (many estimated) and lists the Log Ps, calculated using the chemicalize server (https://chemicalize.com) for tabun, sarin, soman, VX, and some of the structures of the A-series reported by Mirzayanov [3], Hoenig [12], and Ellison [14]. The low vapor pressures, similar to tabun, reported for A-230, A-232, and A-234 [3], suggest that they are moderately persistent in the environment, while the estimated values of Log P suggest a considerable lipophilicity, which will increase the absorption by the body. According to Ellison [14], these compounds can be hydrolyzed to produce HF, HCl, HCN, and highly toxic oximes.

## 7. Toxicity of A-series Nerve Agents

According to Mirzayanov [3] and some other literature sources [6,14], A-230 was found to be 5–8 times more toxic than RVX, which is similar in toxicity to VX, while A-232 presented a toxicity 10 times higher than soman. He also reported that A-242 and A-262 are “ultra-highly” toxic derivatives of A-230 and A-232, but did not define this term neither compared it to the toxicity of any other agent. Considering this information, we estimated the values of LCt_50_ and LD_50_ for these compounds reported in Table 2. Mirzayanov [3] did not report the toxicity of A-234, but considering its structural similarity to A-232, we assumed that it would have similar toxicity. Based on these considerations, we can tell that A-230, A-232, and A-234 are the most toxic nerve agents known. Speculating about A-242 and A-262 toxicity, if Mirzayanov [3] meant that “ultra-highly toxic” is more toxic than A-230 and A-232, these compounds would instead be the most toxic. The fact that they are solids, however, renders them almost useless for weaponization but can make them very attractive for terrorism purposes. As guanidine derivatives, it is very likely that they have good solubility in water and, for example, are perfect to contaminate the water supply.

No antidote is yet reported for the neurotoxics of the A-series, but it is believed that the commercial oximes, like 2-PAM and HI-6, should work against intoxication with these agents [9]. The first reports on experiments in this direction are eagerly anticipated.

## 8. Protection, Treatment, and Decontamination of A-Series Agents

There is no information available yet on protection and decontamination against the A-series agents. However, it is believed that no improvement will be needed in the protective wear currently in use to face these agents. In regards to decontamination, it is believed that, based on their structural similarities to other nerve agents, the A-series compounds can be destroyed by basic solutions with more than 10% by weight of NaOH or NaCO_3_, or undiluted household bleach. It is also speculated that reactive oximes, such as potassium 2,3-butanedione monoximate and basic peroxides, should rapidly detoxify these compounds.

It has been reported that the compounds of the A-series should act in the same way as the nerve agents of the G-series and V-series, by inhibiting the enzyme acetylcholinesterase (AChE) and triggering cholinergic syndrome [17,18,19]. Therefore, preferential prophylaxis would involve the administration of a cocktail containing: (1) an anticholinergic, to reduce the concentration of ACh; (2) an anticonvulsant, to combat the effects of the cholinergic syndrome; and (3) an antidote (usually an oxime) to reactivate AChE. However, it is speculated that the oximes currently in use could be non-effective for these compounds because the intermediate formed during the interaction of these compounds with AChE would be hard to reactivate [17]. This points to bioscavengers as an alternative option for the treatment of intoxication by the A-series agents [17].

## 9. Final Remarks

The A-series of nerve agents represents today a matter of concern in the field of CWs. There are many uncertainties on this issue that still need to be clarified. An urgent, complete, and undoubtful elucidation of their structures and properties is essential at this moment for a better understanding of the real risks posed by these compounds and for the development of effective protection. The knowledge of the correct structures will make it possible for theoretical and experimental investigations towards the discovery of the most appropriate antidotes. Also, its synthesis will allow the evaluation of its physical-chemical properties and toxicology, and it will allow the development of biological and analytical protocols for the identification of its misuse. We believe that this review will bring light to this issue, and it will stimulate researchers to start investigations on this matter, accelerating the process of mastering all aspects related to the A-series nerve agents.

## Figures and Tables

**Table 1:** Physical-chemical properties of some nerve agents [2,3,7,17].

Nerve Agent	Melting Point (°C)	Boiling Point (°C)	Vapor Pressure (Pa)	Solubility at 25 °C (g/L)	Log P
Tabun	−50	240	0.057	9.8	−
Sarin	−57	147	2.9	Miscible	−
Soman	−42	167–200	0.4	2.1	−
VX	Below −51	298	0.0007	Miscible <2.4 °C	−
A−230	5.56	259.92	2.13	4.826	2.14
A−232	5.65	266.59	1.48	1.775	2.55
A−234	3.06	264.11	1.7	0.6512	2.97
A−242	21.46	284.85	0.579	1000	0.45
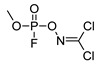	−12.33	218.93	18.5	16.74	1.76
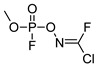	−35.05	191.37	73.8	37.64	1.47
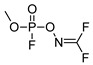	−58.28	162.02	304	84.02	1.18
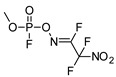	48.34	256.81	1.53	9.940	1.34
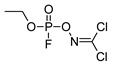	−1.27	237.51	7.04	5.374	2.12
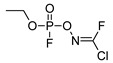	−23.2	211.21	27.4	12.15	1.83
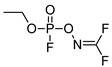	−46.48	183.14	110	27.29	1.54
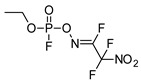	58.85	273.49	0.509	3.157	1.70

**Table 2:** Toxicological data of some nerve agents [6,14].

Neurotoxic Compounds	LCt_50_ for Humans (mg-min/m^3^)	LD_50_ (g/70 kg man)
GA	400	1
GB	55	1.7
GD	70	0.35
VX	15	0.006–0.01
A-230	1.9–3 *	7.5 × 10^−4^–0.002 *
A-232	7 *	0.035 *
A-234	7 *	0.035 *

* Estimated values.

## Abbreviations

AChE	Acetylcholinesterase
CW	Chemical Weapons
CWC	Chemical Weapons Convention
DEAT	2-(diethylamino)ethanethiol
DF	Methylphosphonic difluoride
GA	Tabun
GB	Sarin
GB-2	Binary GB
GD	Soman
GD-2	Binary GD
GOSNIIOKhT	Institute for Organic Chemistry and Technology in Moscow
IJMS	International Journal of Molecular Sciences
IPA	Isopropylamine
LCt_50_	Median Lethal Concentration
LD_50_	Median Lethal Dose
OCAD	OPCW Central Analytical Database
OP	Organophosphate
OPCW	Organization for Prevention of Chemical Weapons
PCF	Methyl phosphorocyanidofluoridate
PCN	2-methylpropyl methylphosphonocyanidate
QL	*O*-Ethyl *O*-2-diisopropylaminoethyl methylphosphonite
NNDA	*N*,*N*-diethyl-2-iminopropan-1-amine
RVX	Russian VX
SLUDGEM	Salivation, Lachrymation, Urination, Defecation, Gastrointestinal, Emesis and Miosis
TEG	1,1,3,3 tetraethylguanidine
TMG	1,1,3,3 tetramethylguanidine
UK	United Kingdom
USA	United States of America
USSR	Union of Soviet Socialist Republics
VX-2	Binary VX
